# A Network Pharmacology Approach for Uncovering the Osteogenic Mechanisms of *Psoralea corylifolia* Linn

**DOI:** 10.1155/2019/2160175

**Published:** 2019-11-07

**Authors:** Luna Ge, Kai Cheng, Jinxiang Han

**Affiliations:** ^1^Shandong Medicinal Biotechnology Center, Key Laboratory for Biotech-Drugs of National Health Commission, Shandong First Medical University and Shandong Academy of Medical Sciences, Jinan 250062, China; ^2^Shandong Cancer Hospital and Institute, Shandong Academy of Medical Sciences, Shandong Cancer Hospital Affiliated to Shandong University, Jinan, Shandong, China

## Abstract

**Background and Aim:**

*Psoralea corylifolia* Linn (PCL) is an herb that is commonly used for alleviating osteoporosis and vitiligo. Although accumulating evidence has demonstrated the antiosteoporotic effect of PCL, the identities of the osteogenic compounds in PCL and their functional targets remain elusive. To investigate the osteogenic ingredients in PCL and their functional mechanisms, network pharmacology analysis was performed on the targets of PCL and osteogenesis.

**Methods:**

The active components of PCL were screened by literature review. The potential protein targets of the active PCL components were predicted with the Traditional Chinese Medicine Systems Pharmacology Database and Analysis Platform (TCMSP), Search Tool for Interactions of Chemicals (STITCH), SwissTargetPrediction, and PubChem. The target networks related to PCL and osteogenic differentiation were constructed by using Cytoscape. MC3T3-E1 cells were used to verify the targets.

**Results:**

Twenty-three active components of PCL and 162 potential target proteins were identified. Further analysis reduced the number of potential target proteins to 71. Of the 23 components, bavachalcone, psoralen, bavachinin, neobavaisoflavone, methoxsalen, psoradin, bakuchiol, and angelicin may be the main active components of PCL that promote bone formation. PPAR*γ* and aryl hydrocarbon receptor (AhR) were verified as targets of PCL in MC3T3-E1 cells, and the western blot and immunofluorescence staining results showed that compared to the control, PCL reduced the expression of these targets.

**Conclusions:**

The active components of PCL and the mechanisms by which they promoted osteogenic differentiation were successfully identified using network pharmacology.

## 1. Introduction

*Psoralea corylifolia* Linn (PCL) is commonly used in clinical Chinese medicine. Modern pharmacology shows that PCL has effects including promoting cardiac health; vascular dilatation; and antitumor, antibacterial, and antiworm properties. Additionally, PCL is used to treat local hair loss, inflammation, vitiligo, leprosy, psoriasis, and eczema [1]. The main active components of PCL are coumarin, flavonoids, and flax flavonoids [2]. PCL and some of its active components have been shown to promote osteogenic differentiation during bone metabolism and can be used as an intervention for osteoporosis [3]. However, the specific molecular mechanism of PCL that promotes bone formation remains unclear.

Traditional Chinese medicine (TCM) is characterized by having multiple targets and multiple effects, and the traditional model of “one-drug, one-target” seriously restricts our ability to scientifically explain the mechanisms of TCM in the treatment of diseases [4, 5]. Network pharmacology was first proposed by Hopkins in 2007, and this method establishes a network that maps the action of a drug, including the disease and drug targets [6] Network pharmacology aims to study the complex relationships among targets, drugs, diseases, and pathways. This technique is novel in drug research and has been proven to be effective in the identification of new active components of TCM and their mechanisms of action [7].

**Figure 1:**
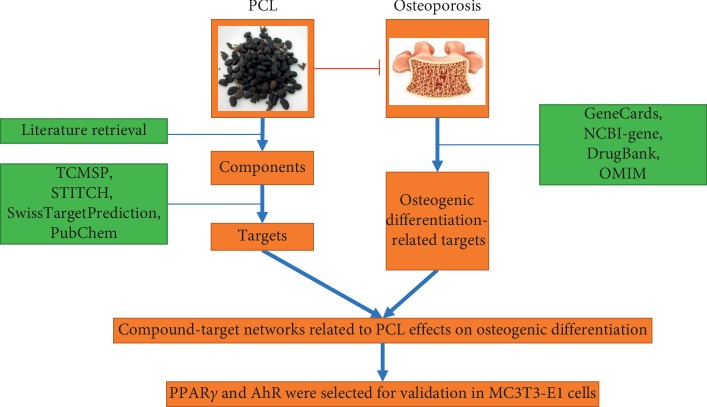
The flowchart of this study.

In this study, network pharmacology was used to identify the main active ingredients of PCL and the mechanisms by which they promoted osteogenic differentiation. Then, we selected aryl hydrocarbon receptor (AhR) and PPAR*γ* to perform experimental verification of the network. A flowchart of this study is depicted in Figure 1.

## 2. Materials and Methods

### 2.1. Materials

PCL was purchased from Beijing Heyanling Pharmaceutical Development Co. Ltd. (Beijing, China) and had been identified as the Genuine Medicinal Herb by Professor Lei Yan in Shandong First Medical University and Shandong Academy of Medical Sciences. A BCIP/NBT Alkaline Phosphatase Color Development Kit, DAPI Staining Solution, Blocking Buffer for Immunol Staining, Antifade Mounting Medium, a bicinchoninic acid (BCA) protein assay kit, and penicillin-streptomycin were purchased from Beyotime (Shanghai, China). A ReverTra Ace® Qpcr RT Kit and Taq SYBR® Green Qpcr Premix were purchased from TOYOBO (Shanghai, China). *β*-Glycerophosphate, P-nitrophenyl phosphate, and ascorbic acid-2-phosphate were purchased from Sigma-Aldrich (St. Louis, MO, USA). TRIzol was obtained from Invitrogen (Carlsbad, CA, USA). The anti-GAPDH, anti-PPAR*γ*, anti-AhR, and CoraLite488-conjugated Affinipure Goat Anti-Rabbit IgG (H + L) antibodies were obtained from Proteintech Group (Wuhan, China). Alizarin red stain was obtained from Cyagen Biosciences (Guangzhou, China). The primers were synthesized by BGI (Shenzhen, China).

### 2.2. Cell Culture

The MC3T3-E1 (subclone 14) osteoblasts cell line was purchased from the Cell Bank of the Type Culture Collection of the Chinese Academy of Sciences (Shanghai, China). The cells were cultured in *α*-MEM (HyClone, Logan, UT, USA) containing 10% fetal bovine serum (Gibco, Waltham, MA, USA), 100 units/mL penicillin and 100 *μ*g/mL streptomycin in 5% CO_2_ at 37°C. Osteogenic induction medium was *α*-MEM supplemented with ascorbic acid-2-phosphate (50 *μ*g/mL) and *β*-glycerophosphate (10 mM).

### 2.3. Alizarin Red and Alkaline Phosphatase (ALP) Staining

Alizarin red and ALP staining were performed as previously described [8]. Osteoblasts cultured in 24-well plates were washed with PBS twice and fixed with 4% paraformaldehyde for 30 min at room temperature. Alizarin red strains were prepared with 0.5% Alizarin red S solution (w/v, pH4.2), and ALP stains were performed with a BCIP/NBT alkaline phosphatase color development kit. Images of stained samples were captured with a digital camera.

### 2.4. ALP Activity Measurement

The BCA protein assay kit was used to detect the total protein concentration. P-nitrophenyl phosphate (pNPP) was used as a substrate to measure the ALP activity. Then, the absorbance was measured at 405 nm using a microplate reader and normalized to the total protein concentration.

### 2.5. RT-qPCR

MC3T3-E1 cells seeded in 6-well plates were cultured with PCL (0, 40, or 80 *μ*g/mL). Total RNA was extracted using TRIzol reagent according to the manufacturer's instructions. Total RNA samples were reverse transcribed into first-strand cDNAs using a ReverTra Ace qPCR RT kit (Toyobo, Osaka, Japan). Quantitative real-time PCR (qRT-PCR) was performed on a LightCycler® 480II real-time PCR system (Roche, Mannheim, Germany) using cDNA as the templates. The condition of amplification reactions was as follows: 94°C for 3 min (initial denaturation), 40 cycles of 15 s denaturation at 94°C, and annealing and extension 64°C for 1 min. All reactions were carried out in triplicate. mRNA expression level was calculated by the 2^–ΔΔCq^ method with normalization to the GAPDH mRNA level [9]. The PCR primer sequences are listed in Table 1.

### 2.6. Western Blot Analysis

Osteoblasts seeded in 75°cm^2^ culture bottles were lysed with cell lysis buffer for western blotting. The protein concentration was measured by the BCA assay kit. Protein samples (40 *μ*g/lane) were separated by SDS-PAGE and electrophoretically transferred to a polyvinylidene fluoride (PVDF) membranes (0.45 *μ*m). The PVDF membranes were blocked in 5% skim milk for 1 h at room temperature and then were incubated at 4°C overnight with the primary antibodies against PPAR*γ*, AhR, and GAPDH. Subsequently, the membranes were washed three times with TBST and incubated with goat anti-rabbit IgG-HRP secondary antibody for 1 h at room temperature followed by three wishes with TBST. Blots were visualized using an enhanced chemiluminescent substrate (ECL) kit. The protein bands were quantified using Image J software and were normalized to the density of the respective GAPDH band.

### 2.7. Immunochemical Staining

Osteoblasts seeded in 48-well plates were fixed with 4% paraformaldehyde at room temperature for 15 min followed by three washes with PBS. Cells were permeabilized with 0.3% Triton X-100 for 30 min and blocked with the blocking buffer (Beyotime, Shanghai, China) for 1 h at room temperature. Samples were incubated overnight at 4°C with primary antibody against AhR (1 : 100, Proteintech). The next day, the samples were rewarmed at 37°C for 1 h and incubated with an Alexa-Fluor 488-conjugated secondary antibody (1 : 500, Proteintech) for 50 min at 37°C and then stained with DAPI (Beyotime, Shanghai, China). After three washes, the images were obtained by a laser scanning confocal microscope (Olympus, Tokyo, Japan).

### 2.8. Preparation of the PCL Water Decoction

The PCL water decoction was prepared as follows: 50 g of PCL was mixed with 10 volumes of water, soaked for 2 h, brought to a boil, and decocted gently for 60 min, then the liquid was removed, and the sample was decocted again in distilled water for a total of 3 times. The three decoctions were combined, heated at 100°C, and concentrated to 250 mL, and the maximum lifetime dosage was 200 mg/mL. Disposable sterile 0.45°*μ*m filters were used to filter the decoction at 4°C.

### 2.9. Database Construction

#### 2.9.1. Compound Database Construction

PubMed, Zhiwang, Wanfang, and other databases were searched for the effective components of PCL. The components were sorted and input into the PubChem database. The chemical structure, formula, Smiles file, molecular weight, PubChem CID, and other documents regarding each component were retrieved, and the required parameters and documents were prepared for target prediction.

#### 2.9.2. Protein Target Database Construction

The Traditional Chinese Medicine Systems Pharmacology Database and Analysis Platform (TCMSP, http://5th.tcmspw.com/tcmsp.php), STITCH (http://stitch.embl.de/), SwissTargetPrediction (http://www.swisstargetprediction.ch/), and PubChem (https://pubchem.ncbi.nlm.nih.gov/) databases were used to predict the targets of PCL. Duplicates were deleted after combining the targets of each component.

#### 2.9.3. Osteogenic Target Database Construction

Relevant targets for osteogenic differentiation were identified by searching the GeneCards, NCBI-gene, DrugBank, and OMIM databases with the following keywords: “osteogenic differentiation,” “osteoblast differentiation,” “osteogenesis,” and “osteogenic.” The results were summarized and sorted according to the gene names.

### 2.10. Bioinformatics Analysis

#### 2.10.1. Gene Ontology (GO) Enrichment Analysis

Dynamic GO enrichment analysis was performed on the identified targets using the OmicShare online tool.

#### 2.10.2. Compound-Target Network Construction and Analysis

The predicted targets of the PCL components were compared with the targets related to osteogenic differentiation, and the overlapping targets were identified with a Venn diagram. These overlapping targets are the targets (effective targets) of PCL that are related to osteogenic differentiation. Cytoscape was used to construct the network structure of the active components and active targets.

### 2.11. Statistical Analysis

SPSS 19.0 was used for the statistical analysis of the experimental data. A *T* test was used to compare data between two groups, and one-way ANOVA was conducted followed by Tukey's post hoc test for multiple comparisons if necessary. In all cases, *p* < 0.05 was considered significant.

## 3. Results

### 3.1. Osteogenic Effect of PCL on MC3T3-E1 Cells

**Figure 2:**
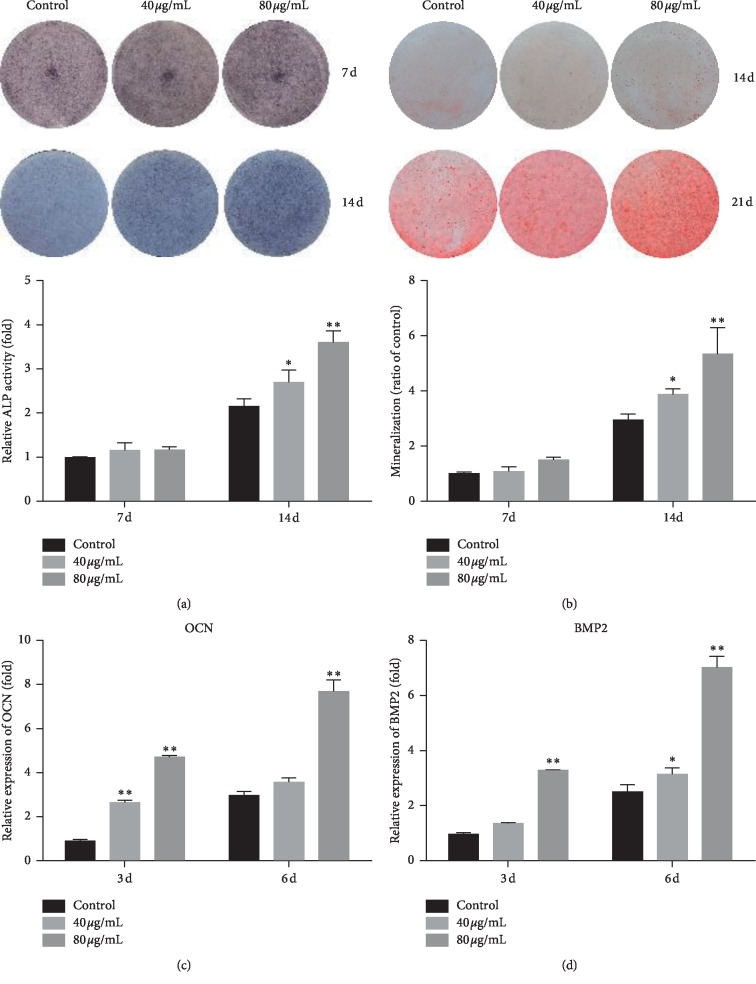
The osteogenic effect of PCL on MC3T3-E1 cells. ALP staining (a) and Alizarin red staining (b) were carried out at 7 d and 14 d after treatment with PCL, respectively. Mineralized nodules were dissolved in cetylpyridine for relative quantification. Total RNA was extracted at 3 d and 6 d after treatment with PCL, and OCN (c) and BMP2 (d) expression was detected by RT-qPCR. ^*∗*^*P* < 0.05, ^*∗∗*^*P* < 0.01.

To verify the molecular mechanism of PCL that promotes bone formation, MC3T3-E1 cells were used as a cellular model for a series of experiments. We confirmed the osteogenic effect of PCL on MC3T3-E1 cells. The results of ALP staining at 7 d and 14 d showed that PCL could significantly promote ALP activity (Figure 2(a)) compared to that in the control. The results of Alizarin red staining at 7 d and 14 d showed that PCL could significantly promote the formation of mineralized nodules (Figure 2(b)) compared to that in the control. At the same time, osteogenic gene expression was also detected in cells that had been treated with PCL. The results showed that PCL significantly promoted the transcriptional levels of OCN (Figure 2(c)) and BMP2 (Figure 2(d)) in a dose- and time-dependent manner compared to those in the controls.

### 3.2. Construction of a Compound Database

To obtain comprehensive information on the targets of PCL, we first identified the ingredients of PCL. As shown in Table 2, 23 ingredients were identified through the literature review. Several of these compounds have been shown to contribute to bone formation, such as psoralen and angelicin.

### 3.3. Construction of a Protein Target Database

**Figure 3:**
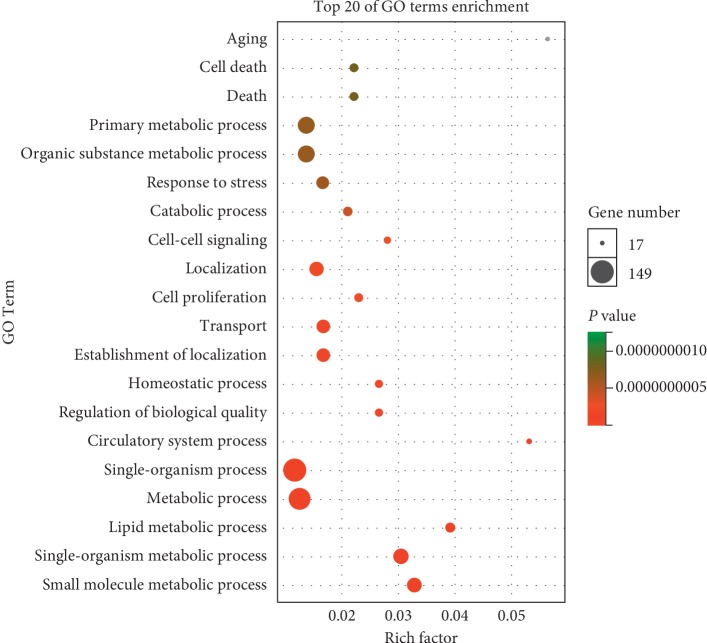
Dynamic GO enrichment analysis.

The targets of the 23 ingredients were predicted with TCMSP, STITCH, SwissTargetPrediction, and PubChem. A total of 162 targets were identified with these databases. To analyze the functional characteristics of these targets, dynamic GO enrichment analysis was performed, and the top 20 GO entries were selected. The results (Figure 3) showed that these targets participate in aging, cell death, primary metabolic processes, and other physiological processes.

### 3.4. Compound-Target Network Construction and Analysis

**Figure 4:**
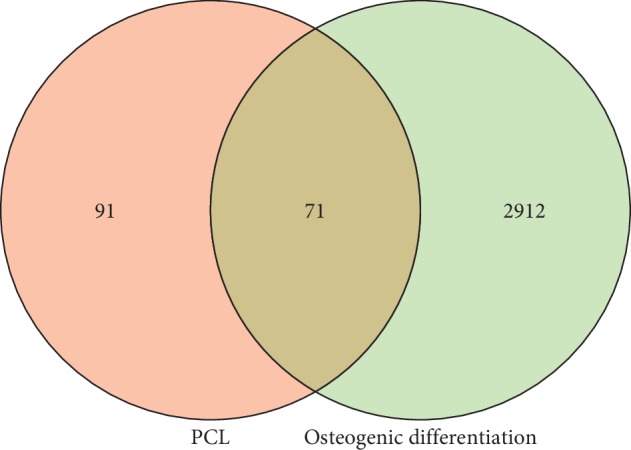
The Venn diagram of the targets of PCL and osteogenic differentiation.

**Figure 5:**
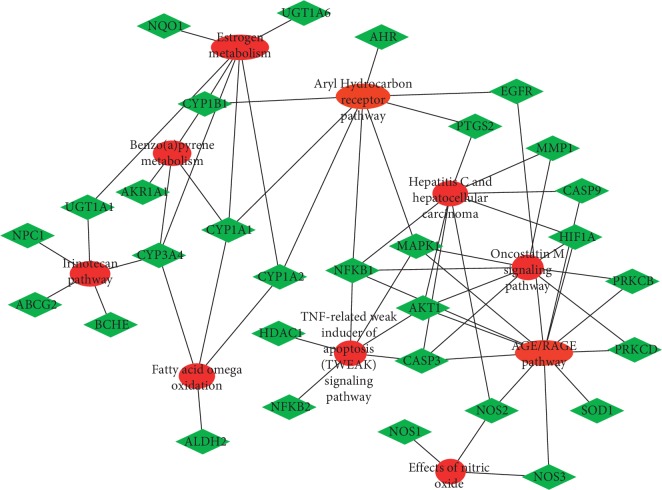
Major pathways involved by the 71 osteogenic differentiation-related targets of PCL.

A total of 2,983 osteogenic differentiation-related targets were obtained from the GeneCards, NCBI-gene, DrugBank, and OMIM databases. The shared targets of PCL and osteogenic differentiation were identified by generating a Venn diagram (Figure 4). There were 71 osteogenic differentiation-related targets of PCL. The enrichment analysis of the 71 targets was performed using Enrichr [10]. The top 10 pathways were imported into Cytoscape for visualization. These targets were primarily involved in the AGE/RAGE pathway, estrogen metabolism, and the AhR pathway (Figure 5).

**Figure 6:**
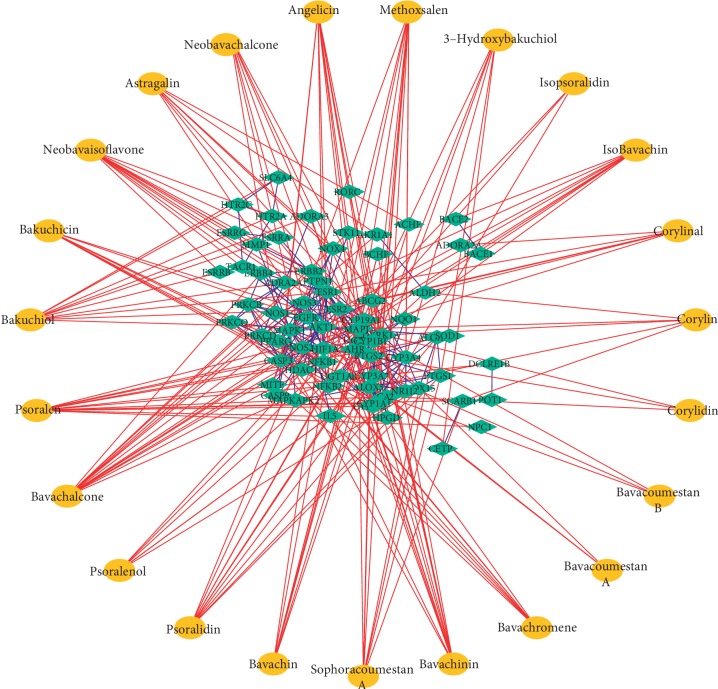
Compound-target networks related to the effects of PCL on osteogenic differentiation.

**Figure 7:**
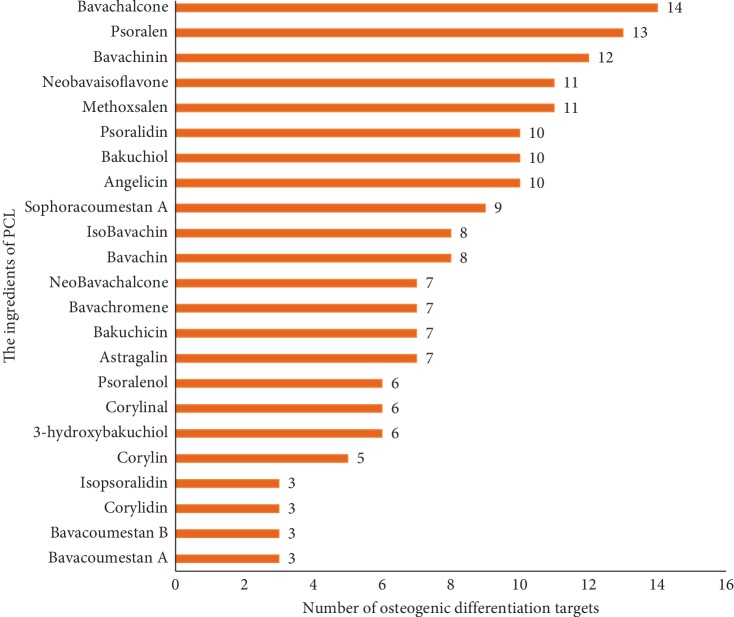
The number of targets of PCL components that participated in osteogenic differentiation.

Compound-target networks related to the effects of PCL on osteogenic differentiation were constructed by using Cytoscape. As shown in Figure 6, the compounds in PCL did not all have the same effects on osteogenic differentiation, and the targets of the compounds varied. Of the 23 components, bavachalcone, psoralen, bavachinin, neobavaisoflavone, methoxsalen, psoradin, bakuchiol, and angelicin had the most targets (Figure 7). These components may be the main active constituents of PCL that are involved in promoting bone formation.

### 3.5. Validation of PPAR*γ* and AhR

**Figure 8:**
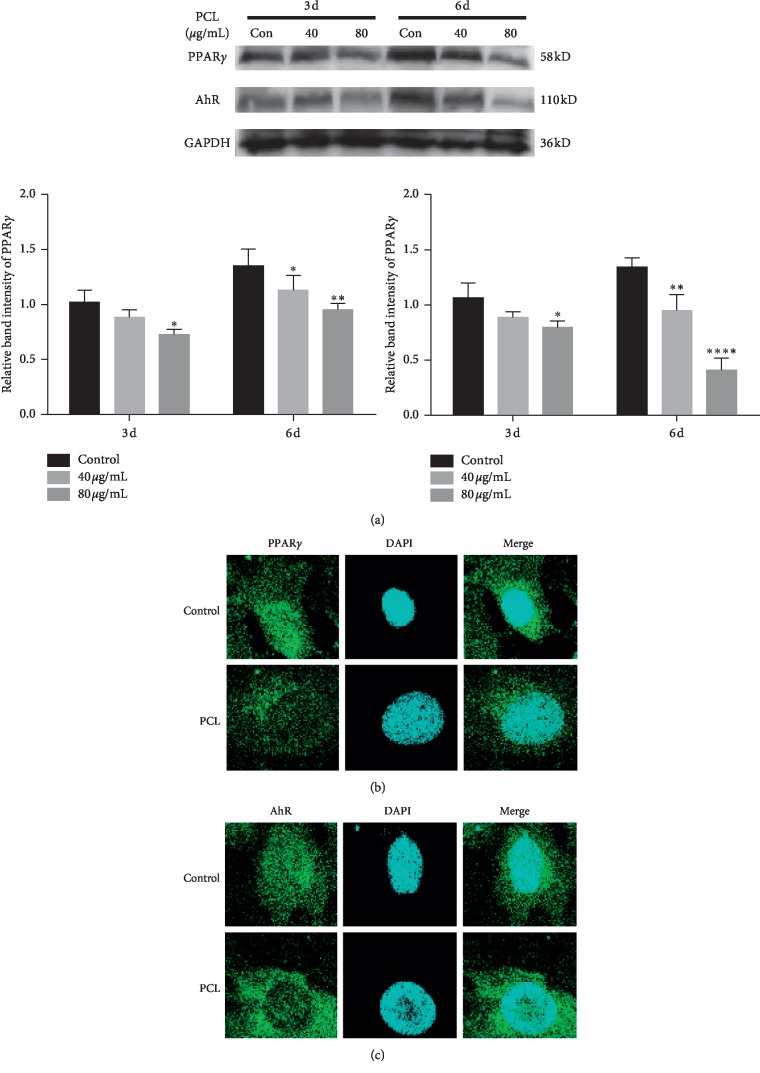
Protein expression of PPAR*γ* and AhR after treatment with PCL. (a) Total protein was extracted at 3 d and 6 d after treatment with PCL, and the relative expression levels of PPAR*γ* and AhR were detected by western blotting. (b) Cell immunofluorescence staining was performed after treatment with PCL for 24 h; ^*∗*^*P* < 0.05, ^*∗∗*^*P* < 0.01, ^*∗∗∗∗*^*P* < 0.0001.

To verify whether the targets that were predicted by network pharmacology promoted osteogenic differentiation in vitro, we selected PPAR*γ* and AhR for verification. MC3T3-E1 cells were treated with 40 *μ*g/mL and 80 *μ*g/mL PCL during the osteogenic induction stage. Western blot results (Figure 8(a)) showed that compared to the control, PCL inhibited the expression of PPAR*γ* and AhR at 3 d and 6 d, respectively. To investigate the cellular distribution of PPAR*γ* and AhR, cell immunofluorescence staining was performed. As shown in Figure 8(b), compared to the control, PCL mainly reduced the expression of PPAR*γ* and AhR in the nucleus.

## 4. Discussion

TCMs are composed of many components, and their mechanism of action involves a variety of target proteins and pathways. PCL is a common herb in TCM prescriptions and is frequently used in the treatment of osteoporosis. Osteoporosis occurs as the result of an imbalance between bone resorption and bone formation in the human body [11]. This disease can be treated by inhibiting bone resorption and promoting bone formation, but most of the drugs for osteoporosis promote bone formation. In this study, we demonstrated that PCL could effectively promote bone formation in MC3T3-E1 cells (Figure 2). However, the specific mechanism through which PCL promotes osteogenic differentiation remains unclear. Network pharmacology based on various bioinformatics methods was used in the present study to examine the network of the molecular mechanisms of PCL.

In this study, we first identified the active components of PCL. Through literature review, 23 components of PCL were identified, and their molecular weights are shown in Table 2. Some of these components, such as psoralen and angelicin, have been shown to promote osteogenic differentiation. Then, the targets of the 23 components were predicted with the TCMSP, STITCH, SwissTargetPrediction, and PubChem databases. A total of 162 targets were predicted, and these targets participated in aging, cell death, primary metabolic process, and other physiological processes (Figure 3). To confirm whether all 162 targets were related to osteogenic differentiation, we searched for osteogenic differentiation targets using the GeneCards, NCBI-gene, DrugBank, and OMIM databases. With these databases, we identified 2,983 targets of osteogenic differentiation. By examining which genes were shared by these two gene sets, 71 of the 162 targets of PCL were found to be involved in osteogenic differentiation (Figure 4). Enrichment analysis showed that these targets were primarily involved in the AGE/RAGE pathway, estrogen metabolism, and AhR pathway. Estrogen metabolism plays an important role in skeletal health [12]. Estrogen-deficient osteoporosis can be caused by estrogen deficiency or an estrogen synthesis disorder. PCL may regulate estrogen metabolism through UGT1A6, CYP1B1, CYP1A1, and other gene products.

Cytoscape was used to construct compound-target networks related to the effects of PCL on osteogenic differentiation. Of the 23 components in PCL, bavachalcone, psoralen, bavachinin, neobavaisoflavone, methoxsalen, psoradin, bakuchiol, and angelicin have the most targets (Figure 6). These components may be the main active constituents of PCL that promote bone formation. In our previous studies on angelicin, we found that angelicin could promote osteogenic differentiation in vitro by targeting AhR [8]. In addition to participating in the AhR pathway, AhR can also participate in estrogen metabolism by regulating CYP1A1 [13]. Angelicin also prevented osteoporosis by downregulating the PPAR*γ* protein [14]. At the same time, psoralen aided in the rehabilitation of the steroid-induced avascular necrosis of the femoral head by reducing the expression of PPAR*γ* [15]. All these studies have proved that it is feasible to use network pharmacology to predict complex targets of TCM.

To verify whether PCL promoted osteogenic differentiation by decreasing the expression of AhR and PPAR*γ*, MC3T3-E1 cells were treated with PCL during osteogenic induction. Western blot results showed that compared to the control, 40 *μ*g/mL and 80 *μ*g/mL PCL significantly decreased the expression of PPAR*γ* and AhR. Cell immunofluorescence staining showed that PCL mainly reduced the expression of PPAR*γ* and AhR in the nucleus.

## 5. Conclusion

As a multicomponent TCM, PCL has multiple targets and multiple effects. To identify the main active components of PCL and to clarify the mechanism through which PCL promoted osteogenic differentiation, network pharmacology was used. Twenty-three components of PCL were identified through literature review. A total of 71 osteogenic differentiation-related targets of PCL were predicted. PPAR*γ* and AhR were selected for further experimental verification. Western blotting and immunofluorescence staining results showed that compared to the control treatment, PCL reduced the protein expression of PPAR*γ* and AhR. These findings can inform future research on the clinical applications of PCL, and the network analysis method that was used here is amenable to the study of other TCMs.

## Figures and Tables

**Table 1:** Primer sequences.

Name	Sequences
OCN-FP	AATGAGGTCACATCCATCCTG
OCN-RP	CACCCGAGTGGTAGTCACAA
BMP2-FP	ACAGAGCTATTAAAGTGACAGTGGAC
BMP2-RP	GGCGATCAGAGAACAAACTAGG
GAPDH-FP	TGTCCGTCGTGGATCTGAC
GAPDH-RP	CCTGCTTCACCACCTTCTTG

**Table 2:** The components of PCL.

No.	PubChem CID	Molecule name	MW
1	6199	Psoralen	186.166
2	10658	Angelicin	186.166
3	4114	Methoxsalen	216.20
4	3083848	Bakuchicin	186.166
5	5321800	Bavachromene	322.36
6	5281806	Psoralidin	336.343
7	12304285	Isopsoralidin	336.343
8	5316096	Corylidin	368.341
9	5321811	Bavacoumestan A	352.342
10	5321820	Bavacoumestan B	352.342
11	14630492	Sophoracoumestan A	334.327
12	5282102	Astragalin	448.38
13	5316097	Corylin	320.344
14	5320053	Neobavaisoflavone	322.36
15	44257227	Corylinal	282.251
16	5320772	Psoralenol	338.359
17	14236566	Bavachin	324.376
18	193679	Isobavachin	324.376
19	6450879	Bavachalcone	324.376
20	10337211	Bavachinin	338.403
21	5320052	Neobavachalcone	298.294
22	5468522	Bakuchiol	256.389
23	56833075	3-Hydroxybakuchiol	272.388

## Data Availability

The data used to support the findings of this study are available from the corresponding author upon request.
